# Intergeneric Relationships within the Family Salicaceae *s.l.* Based on Plastid Phylogenomics

**DOI:** 10.3390/ijms20153788

**Published:** 2019-08-02

**Authors:** Meng-Meng Li, De-Yan Wang, Lei Zhang, Ming-Hui Kang, Zhi-Qiang Lu, Ren-Bin Zhu, Xing-Xing Mao, Zhen-Xiang Xi, Tao Ma

**Affiliations:** 1 Key Laboratory of Bio-Resource and Eco-Environment of Ministry of Education, College of Life Sciences, Sichuan University, Chengdu 610065, China; 2 CAS Key Laboratory of Tropical Forest Ecology, Xishuangbanna Tropical Botanical Garden, Chinese Academy of Sciences, Mengla 666303, China

**Keywords:** Salicaceae, phylogenetic relationship, plastid genome, comparative genomics, repeat sequences

## Abstract

Many Salicaceae *s.l.* plants are recognized for their important role in the production of products such as wood, oils, and medicines, and as a model organism in life studies. However, the difference in plastid sequence, phylogenetic relationships, and lineage diversification of the family Salicaceae *s.l.* remain poorly understood. In this study, we compare 24 species representing 18 genera of the family. Simple sequence repeats (SSRs) are considered effective molecular markers for plant species identification and population genetics. Among them, a total of 1798 SSRs were identified, among which mononucleotide repeat was the most common with 1455 accounts representing 80.92% of the total. Most of the SSRs are located in the non-coding region. We also identified five other types of repeats, including 1750 tandems, 434 forward, 407 palindromic, 86 reverse, and 30 complementary repeats. The species in Salicaceae *s.l.* have a conserved plastid genome. Each plastome presented a typical quadripartite structure and varied in size due to the expansion and contraction of the inverted repeat (IR) boundary, lacking major structural variations, but we identified six divergence hotspot regions. We obtained phylogenetic relationships of 18 genera in Salicaceae *s.l.* and the 24 species formed a highly supported lineage. *Casearia* was identified as the basal clade. The divergence time between Salicaceae *s.l.* and the outgroup was estimated as ~93 Mya; *Salix*, and *Populus* diverged around 34 Mya, consistent with the previously reported time. Our research will contribute to a better understanding of the phylogenetic relationships among the members of the Salicaceae *s.l.*

## 1. Introduction

The previously defined Salicaceae *sensu stricto* (*s.s.*) only included *Salix* and *Populus* [1], but later more than 50 genera were classified into the Salicaceae *sensu lato* (*s.l.*) family, containing over 1000 species [2]. The family Salicaceae *s.l.* is a woody shrub plant, ranging in height from less than a few centimeters to tens of meters. The species in Salicaceae *s.l.* are primarily distributed in cold, tropical, and warm temperate regions and occupy extremely varied habitats [2,3]. This family’s sexual systems are highly diverse. Most genera are dioecious, whereas, some are monoecious. Both XY and ZW sex determination systems have been reported in the dioecious species, indicating an amazingly diversified history of sex determination [4,5]. The species of this family have many uses. There are varied chemicals produced by the family. An abundant oil containing unsaturated fatty acids can be synthesized from *Idesia* fruits [6]. The early medicine, aspirin, was first isolated from willow and poplar bark, and the willow species have been developed as bioenergy crops [7]. *Populus* species have become model organisms for basic research in molecular biology and genetics because of their small genome size and fast growth rates. Moreover, they play important roles in the ecosystem, plant domestication, and conservation, as well as being one of the most economically important groups of forest trees [8].

The plastid genome is widely used in plant genetic population and phylogeny analysis because of its slow rate of nucleotide substitution in gene coding genes and relatively conservative gene structure and content, which can also increase phylogenetic resolution at the lower taxonomic levels [9,10,11,12,13,14]. The plastid genome usually encodes about 80 unique proteins, 30 transfer RNAs (tRNAs), and four ribosomal RNAs (rRNAs) [15,16]. For terrestrial plants with photosynthesis, the plastid genome is 120–220 kb in size and has the typical feature of two inverted repeats (IRs), 20–28 kb in size, separated by small single copies (SSC) and large single copies ( LSC) with sizes of 16–27 kb and 80–90 kb, respectively [17,18]. The size of IR in plastids varies widely for different groups, genus, family or species [13,19,20]. The IR copies recombine themselves in order to maintain or confer stability in the remaining plastome [17,18,21]. With the development of high-throughput sequencing technology in recent years, the number of complete sequenced plastid genomes has increased rapidly [22]. The whole plastid genome of 61 species in Salicaceae *s.l.* has been sequenced and stored in the GenBank.

In previous studies [23,24,25,26], their work mainly focused on the relationship of the main subclades in the genera of *Salix* and *Populus*, because the delimitation of species in these genera remains controversial. There are 29–70 species in the genus *Populus* and based on their morphological characteristics they have been grouped into the following six sections: *Abaso*, *Turanga*, *Populus*, *Leucoides*, *Aigeiros*, and *Tacamahaca* [27]. For the genus *Salix*, about 450 species have been published and two main subclades have been identified [28,29,30]. The most recent study used 42 species from six genera based on the complete plastomes in order to examine phylogenetic relationships of Salicaceae [31]. Although several other genera in Salicaceae were mentioned in their research, their main purpose was to determine the relationship of subclades in the genus of *Salix* and *Populus*. We know little about the phylogenetic relationships of the other 48 genera in Salicaceae *s.l.*

In this study, we sequenced 20 plastid genomes in the family, and added the following four previously published plastid genomes: *Populus euphratica*, *Salix interior*, *Idesia polycarpa maxim*, and *Poliothyrsis sinensis*. In total, there were 24 species from 18 genera of Salicaceae *s.l.*, as well as two outgroups, *Passiflora laurifolia* and *Passiflora ligularis*. We mainly aimed to: (1) determine the repeat sequence variations of plastid genomes, (2) examine structural changes in the plastomes of the Salicaceae *s.l.*, and (3) delimit intergeneric relationships within Salicaceae *s.l.*

## 2. Results

### 2.1. Characteristics of the Plastid Genomes

Twenty complete plastid genomes belonging to fourteen genera of the family Salicaceae *s.l.* were newly generated in this study (Table 1). All of the genome sequences have been submitted to the GenBank. In order to fully display the characteristics of the plastid genomes, we further collected four sequences of other species in the Salicaceae *s.l.* from the NCBI GenBank. In total, our subsequent comparative analysis included 24 species representing 18 genera of the family Salicaceae *s.l.* (Table 1). The total length of the chloroplast genome sequences ranged from 155,144 bp in *Flacourtia ramontchii* to 158,605 bp in *Bennettiodendron brevipes*. The structure of the genomes displayed a typical quadripartite structure, with a pair of inverted-repeat (IR) regions of 27,168–27,926 bp, separated by a large single copy (LSC) of 83,391–86,350 bp and a small single copy (SSC) of 16,305–17,889 bp. The LSC regions exhibited the greatest standard deviation in sequence length (s.d. = 872.58 bp), followed by the SSC regions (s.d. = 470.12 bp) and the the IR regions (s.d. = 217.50 bp). Nucleotide composition with an overall GC content of 36.8% was nearly identical in all plastid genomes.

**Figure 1:**
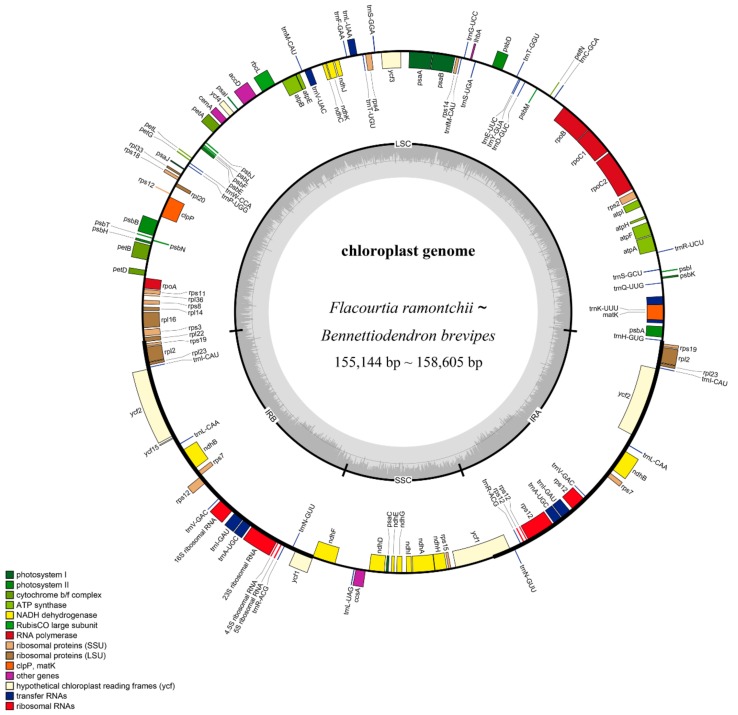
Gene map of 20 Salicaceae *s.l.* chloroplast genomes. Functional categories of genes are color-coded. Genes inside the circle are transcribed clockwise and genes outside the circle are transcribed counter clockwise. The dashed area in the inner circle indicates the GC content of the plastid genome.

A total of 131 functional genes with the same order were annotated in each of the newly sequenced plastomes, of which 102 were unique genes, including 78 protein-coding genes, 30 tRNA genes, and four rRNA genes (Figure 1, Table 2). Most of these genes occurred as a single copy, while 19 genes were duplicated in the IR regions (Table 1). The gene *cemA* contained premature termination codons in *Casearia decandra Jacq* and *Casearia velutina*, while gene *atpF* contained premature termination codons in *Olmediella betschleriana*.

### 2.2. Repeat Sequence Analysis

**Figure 2:**
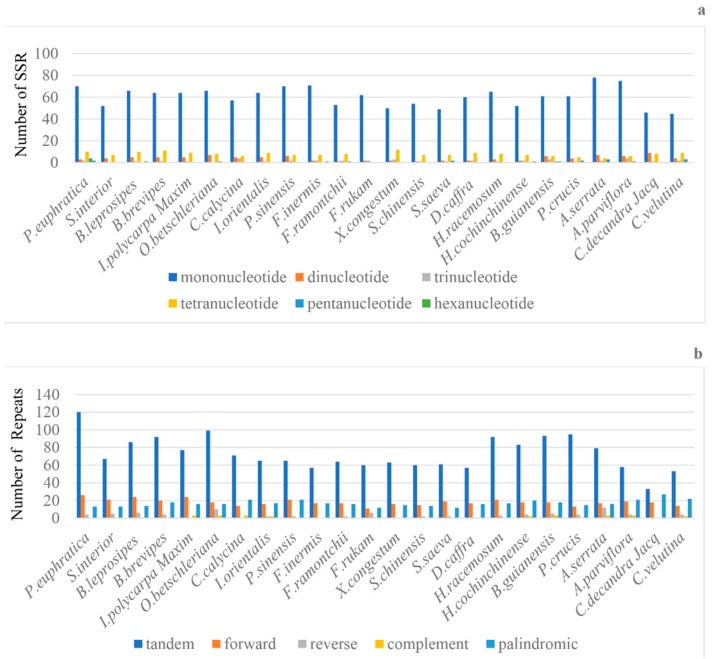
Comparison of repeat sequences among 24 plastomes. The *X*-axis represents the species and the *Y*-axis represents the number of repeats. (**a**) Frequency of selected motifs of simple sequence repeats (SSRs) and (**b**) number of each repeat type: tandem repeats, forward repeats, palindromic repeats, reverse repeats, and complement repeats.

We identified a total of 1798 simple sequence repeat (SSR) loci across the 24 Salicaceae *s.l.* plastids (Figure 2a, Table S1). Of these, 1455 were mononucleotide repeats accounting for about 80.92% of the total SSRs, while 180 (10.01%), 98 (5.45%), 41 (2.28%), 18 (1.00%), and six (0.33%) were tetra-, di-, tri-, penta-, and hex-nucleotides repeats, respectively. The penta- and hex- nucleotides repeats were very rare across the plastid genomes. Most (74.36%) SSR loci were located in the intergenic regions, whereas, 9.07% were in intron and 16.57% were in the protein-coding regions.

We also identified 5 other types of repeats, including 1750 tandems, 434 forward, 407 palindromic, 86 reverse, and 30 complementary repeats (Figure 2b, Table S2, Table S3). Among these, tandem repeats are the most frequent type of repeats (64.65%), while complementary repeats are the least. We found that the tandem repeat sequences were mainly located in non-coding regions, whereas, only a few were located in the coding regions (*ycf2*, *rpl22*, *rpl14*, *rpoC2*, *ycf1*, *ndhD*, *ndhG*, *psbL*, *ndhF*, *rpoA*, *petD*, and *ccsA*). Most of the five types of repeats were concentrated in the intergenic regions (Table S3).

### 2.3. Inverted Repeats and Genome Comparison

**Figure 3:**
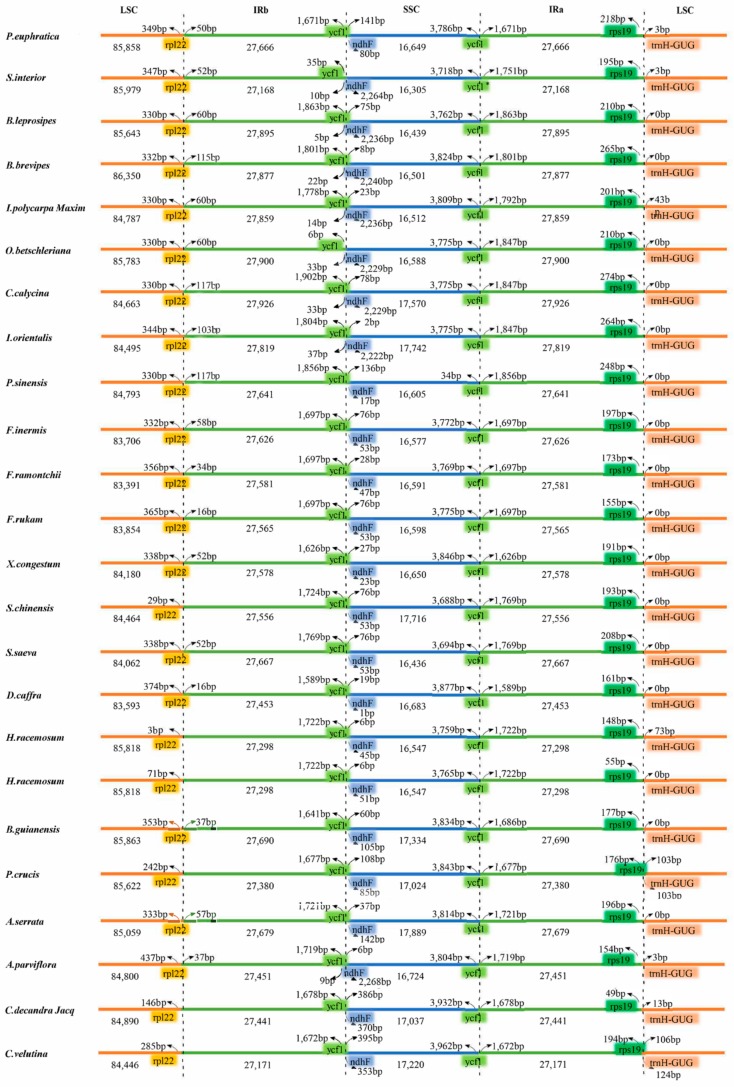
Comparison of the border positions of large single copy (LSC), small single copy (SSC), and inverted repeat (IR) region borders among plastid genomes of 24 Salicaceae *s.l.* species. Complete genes and portions of genes adjacent to the junctions are depicted by differently colored blocks.

Next, we conducted whole genome alignment using the program mVISTA (*Populus euphratica* as reference), and the results showed that both the content and order of the genes were highly conserved in the Salicaceae *s.l.* plastids (Figure S1). The IRa/SSC boundary positions for all species were located in the *ycf1* gene, while the border genes of IRa and LSC were *Rps19* and *trnH-GUG*, respectively. Only slight variations of the border structure were identified across these plastid genomes. For example, the IRb/LSC junction was located within the *rpl22* gene in all but six species (*Scolopia chinensis*, *Homalium racemosum*, *Homalium cochinchinense*, *Prockia crucis*, *Casearia velutina*, and *Casearia decandra Jacq*). The *ndhF* gene was located entirely in the SSC region of 16 species, while in the other eight species it extended into the IRb region (Figure 3).

### 2.4. Divergence Hotspots of Plastid Genomes

**Figure 4:**
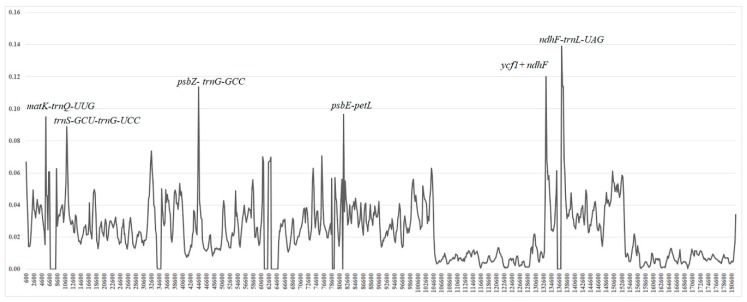
Comparison of the nucleotide variability (Pi) values of the plastomes. *X*-axis: position of the midpoint of a window, *Y*-axis: nucleotide diversity of each window (window length: 600 bp and step size: 200 bp).

To evaluate the level of sequence divergence, we calculated the percentages of variation using a sliding window approach (Figure 4). Across the 24 species, the values of nucleotide variability ranged from 0 to 0.139, with an average of 0.023, suggesting a high conservation of plastid genomes within Salicaceae *s.l.* Six relatively high variable regions (divergence hotspots) were identified, which comprised one gene region (*ycf1 + ndhF*) and the following five intergenic regions: *matK-trnQ-UUG*, *trnS-GCU-trnG-UCC*, *psbZ-trnG-GCC*, and *ndhF-trnL-UAG, psbE-petL*.

### 2.5. Phylogenetic Analysis

**Figure 5:**
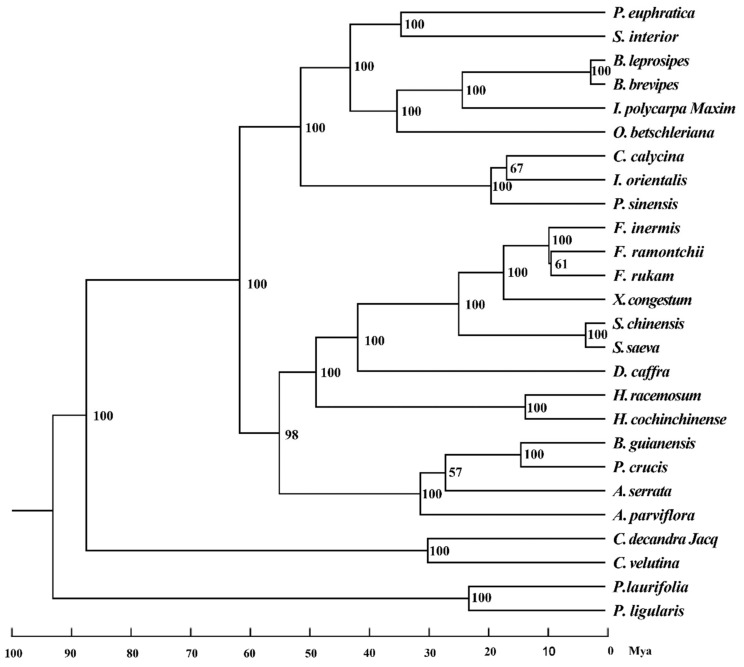
Phylogenetic tree of the 26 species based on 63 protein-coding genes using maximum likelihood (ML). Bootstrap values (1000 replications) between different genera are shown at the nodes. The branches are drawn in proportion to the posterior means of divergence times estimated under the GTR + I + G model with an independent relaxed clock and birth–death sampling, and the outgroup divergence constraint of 87 to 97 Mya.

To obtain an accurate phylogenetic relationship of Salicaceae *s.l.* species, we performed multiple sequence alignments of these 24 complete plastid genomes, including an additional two species from *Passiflora* as the outgroup. The final concatenated dataset, which included 63 protein-coding genes (Table S4) and 51,780 nucleotides, after trimming poorly aligned regions, produced a highly supported topology based on the maximum likelihood (ML) strategy (Figure 5). Two subfamilies in the phylogenetic tree, Samydoideae and Salicoideae, formed a highly supported monophyletic. *Casearia* (*Casearia decandra Jacq* and *Casearia velutina*) was the only genus from Samydoideae in this study and was identified as the basal clade. The remaining species belonged to Salicoideae and were divided into two main clades. Furthermore, we estimated divergence times from the plastome dataset using an approximate likelihood method. The Salicaceae *s.l.* was estimated to diverge from the outgroup around 93 Mya.

## 3. Discussion

### 3.1. Features of Plastid Genomes

We used the information from complete plastid genome sequencing to research and analyze the plastomes of Salicaceae *s.l*. species. Generally, the plastids of angiosperms are considered to be highly conserved, have a typical tetragonal structure, usually contain about 110–130 unique genes, and the genome size, GC composition, as well as gene and intron content are identical in most angiosperms [32,33,34,35]. The shift of IR-SC boundaries was a common evolutionary event and played an important role in plastome size variation [36]. The IR regions of the Salicaceae *s.l.* species varied in size from 27,168 bp to 27,926 bp and the total length ranged from 155,144 bp in *Flacourtia ramontchii* to 158,605 bp in *Bennettiodendron brevipes*, as a result of the expansion and contraction of the IR borders.

Previous studies have revealed that many chloroplast genes, such as *infA*, *rpl22*, *rps19*, *rpl2* intron and *rpl23*, are transferred to the nucleus or lost [37,38,39]. In our study, only *infA* was not found in the plastid genome of Salicaceae *s.l. cemA* and contained premature termination codons in *Casearia decandra Jacq* and *Casearia velutina*, whereas, *atpF* was a pseudogene in *Olmediella betschleriana*. Gene duplication caused by IR is common in plastomes and is believed to be an important driving force in the evolution of genomes, leading to the creation of new genes and new gene functions [40]. Gene duplication has been previously reported in multiple angiosperm lineages and most of them are tRNAs [41,42,43,44,45,46]. Duplication of protein-coding genes outside of the IR is rare. In our results, seven tRNA genes and eight protein-coding genes were duplicated in the inverted-repeat region.

### 3.2. Repeat Sequence Variations

SSRs are a type one to seven nucleotide unit tandem repeat sequence frequently observed in plastid genome and the changes in copy number are usually polymorphic [47,48,49,50]. The SSRs are considered effective molecular markers for plant species identification and population genetics because they exhibit codominant inheritance, high repeatability, and high variation within the same species [47,48,49,50]. In this study, the mononucleotide repeats were the most common, of which 822 were T type accounting for about 57%, while 606 (41%), 16 (1%), 11 (0.7%), were A, C, and G type, respectively. Most of the SSR loci were located in the non-coding region, and only 16% of the SSR loci were found in the gene-coding region. These SSRs were located in 39 coding genes, and the genes with the highest SSR frequency are *ycf1*, *rpoc2*, and *ndhF*, which are 95, 48, and 26 times, respectively (Table S1). The plastome size variation was previously reported to contribute to tandem [36,51] and dispersed repeats [41,52,53,54]. We analyzed the direct repeats, inverted repeats, reverse repeats, complementary repeats, and tandem repeats in 24 Salicaceae *s.l.* species. The analysis showed that the number of tandem repeats is more than the other repeats, while complementary repeats are the least common in these species. Most of the repeats are distributed in the intergenic and intron regions, similar to those reported in other angiosperm lineages [55]. These repeat sequences and plastid SSRs provided molecular markers for studying the genetic diversity, population structure, and phylogenetics of Salicaceae *s.l.*

### 3.3. Comparative Analyses

Comparing chloroplast genomics not only provides insight into chloroplast evolution patterns [56], but also contributes to phylogenetic studies to understand the evolutionary relationships among taxa and apply them to species breeding and conservation [57]. We compared the 24 complete plastid genomes of 18 genera in Salicaceae *s.l.* The species in this family have a conserved plastid genome that lack major structural variations. The main cause of genomic length variation in higher plant plastids is the change in the position of the boundary between IR and SSC/LSC [58]. In this study, the location of the boundary and length of the IR regions showed that variation among the 24 species and the plastid genomes differed slightly in size, with *Bennettiodendron leprosipes*, *Bennettiodendron brevipes*, *Olmediella betschleriana*, *Carrierea calycina*, and *Ito orientalis* larger than other species, which may be related to their IR expansion. IR expansion/contraction also represents a highly variable region, which can be used to study the phylogenetic classification of plants and achieve molecular improvement of plant phenotypes. In addition, the position of the *trnH* gene, which was found in the LSC region of all 24 plastid genomes, was conserved. This result was consistent with the observation that the *trnH* gene is usually located in the IR region in monocots but is found within the LSC region in dicots.

### 3.4. Divergence Hotspot Analysis

The whole aligned sequences revealed relatively low divergence, however, six (*matK-trnQ-UUG*, *trnS-GCU-trnG-UCC*, *psbZ-trnG-GCC*, *ndhF-trnL-UAG*, *psbE-petL*, and *ycf1+ndhF*) displayed high variation. Further work is still necessary to determine whether these six variable loci could be used in phylogenetic analyses of related Salicaceae *s.l.* species or as potential molecular markers for population genetics and phylogenetics.

### 3.5. Phylogenetic Relationships

The deep relationships of mimosoids were poorly resolved by phylogenetic studies applying a few plastid markers [59,60]. Plastid phylogenomics has been proven to be efficient to resolve difficult relationships at the family level such as Orchidaceae [61] and the lower taxonomic level such as subfamilies Bambusoideae [62] and Chloridoideae of Poaceae. For the phylogenetic relationship between the species of Salicaceae, earlier studies focused on Salicaceae *s.s.* Preliminary phylogenetic frameworks for *Salix* and *Populus* have previously been provided [23,24,25,26]. In addition, some studies of angiosperm and Malpighiales also involve the phylogenetic relationships of several genera of Salicaceae *s.l.* [63,64,65,66]. However, phylogenetic analyses focusing on Salicaceae *s.l.* have been limited to the use of one or a few genes obtained from plastid or nuclear genomes [2,67]. In this study, we obtained phylogenetic relationships of 18 genera in Salicaceae *s.l.* using 63 plastid genes from 26 species. According to the Angiosperm Phylogeny Group’s most recent phylogeny, APG IV [2,68], the phylogenetic tree is divided into Salicoideae and Samydoideae. *Casearia* from Samydoideae was identified as the basal clade. The genera in one of the two main clades of Salicoideae included *Populus*, *Salix*, *Bennettiodendron*, *Idesia*, *Olmediella*, *Carrierea*, *Itoa*, and *Poliothyrsis*, all belonging to Saliceae. The other clade was divided into two subclades, one subclade included five genera, *Flacourtia*, *Xylosma*, and *Dovyalis* belonging to the Saliceae, *Scolopia* belonging to Scolopieae, and *Homolium* belonging to Homalieae. The other subclade consisted of four species of three tribes, of which *Banara* and *Prockia* belong to Prockieae, *Abatia* and *Azara* belong to Abatieae and Saliceae, respectively. The Saliceae with the most species is not monophyletic.

We further estimated divergence timescales of the major clades within the Salicaceae *s.l.* according to the calibrations of the species tree constructed on the basis of 63 protein-coding genes. The split between Salicaceae *s.l.* and the outgroup was estimated as ~93 Mya, and the basal *Casearia* was estimated to diverge from other clades around 87 Mya. The clade of Salicoideae is estimated to have originated around 61 Mya. *Salix* and *Populus* diverged around 34 Mya, consistent with the previously reported [26].

## 4. Materials and Methods

### 4.1. Sampling and Genome Sequencing

Fresh leaves and silica-gel dried materials were sampled from 20 species representing 14 genera of the family Salicaceae *s.l*. The voucher specimens for the ten fresh sampled plants collected from XiShuangBanNa Tropical Botanical Garden (Mengla, China) were deposited at the Key Laboratory of Bio-Resource and Eco-Environment of the Ministry of Education (Chengdu, Sichuan, China). The ten silica-gel-dried materials were obtained from Harvard University Herbaria and the Arnold Arboretum of Harvard University. For each species, we used the modified CTAB method [69] to extract the total genomic DNA from dry leaves. About 5 ug purified DNA was used to construct Illumina paired-end libraries with an insert size of 500 bp and sequenced using the HiSeq X Ten System by Novogene (Beijing, China). At least two gigabases (Gb) of 2 × 150 bp short reads data were generated for each sample. Reads with a Phred quality score <7 and more than 10% ambiguous nucleotides were filtered.

### 4.2. Plastid Genome Assembly and Annotation

The filtered reads were assembled by NOVOPlasty v2.7.1 [70], and we used the complete plastid genome *Itoa orientalis* and *Flacourtia indica* (NC_037411.1 and NC_037410.1) as the reference, and then used Geneious v11.1.5 [71] to correct the assemble. Then, we annotated the assembled plastid genome in Plann v1.1 [72]. The positions of starts, stops, introns, and exons were manually adjusted using Sequin v15.50. In addition, the physical map of the plastid genome was generated using OGDRAW v1.2 [73]. The complete plastid genome together with gene annotations was submitted to the GenBank. The accession numbers are shown in Table 1.

### 4.3. Repeat Sequence Analysis

The SSRs were evaluated using the online software MISA (https://webblast.ipk-gatersleben.de/misa/) [74] with thresholds: 10, 5, 4, 3, 3, 3 ten repeat units for mononucleotide SSRs, five repeat units for dinucleotide SSRs, four repeat units for trinucleotide SSRs and three repeat units for tetra-, penta-, and hexanucleotide SSRs. We used the web-based analysis tool REPuter (https://bibiserv.cebitec.uni-bielefeld.de/reputer) [75] to detect the repeat sequences, including reverse, forward (direct), complement, and palindromic (inverted), with a minimal repeat size of 30 bp and Hamming distance less than or equal to 3 (90% or greater sequence identity). Tandem Repeats Finder v4.09 (http://tandem.bu.edu/trf/trf.submit.options.html) [76] was used to determine the trandem repeats, alignment parameters match, mismatch. Indel were set as 2, 7, and 7.

### 4.4. Genome Comparative Analysis

Furter, mVISTA (http://genome.lbl.gov/vista/mvista/submit.shtml) [77], a web-based program, was used to compare similarities and detect any rearrangements or inversions among different plastid genomes. We used this software (*Populus euphratica* as reference) to discover any significant interspecific and intergeneric variations among plastid genome sequences of Salicaceae *s.l.* Additionally, the IR expansion/contraction regions were compared among the 24 Salicaceae *s.l.* species (20 newly generated plastid genomes in this study and 4 previous published plastid genomes: *Populus euphratica*, *Salix interior*, *Idesia polycarpa Maxim*, and *Poliothyrsis sinensis*).

### 4.5. Sequence Divergence Analysis

After aligning all sequences using Mafft v7.313 [78], we located and counted the SNP sites in the plastid genomes using DnaSP v6.0 [79]. The nucleotide variability (Pi) and polymorphic sites (S) were evaluated using a sliding window analysis with a step size of 200 bp and window length of 600 bp.

### 4.6. Phylogenetic Analyses

There were 26 plastid genomes used for phylogenetic analyses, including 2 outgroups (*Passiflora laurifolia* and *Passiflora ligularis*) and 24 Salicaceae *s.l.* species (Table 1). We extracted 63 protein-coding genes in all 26 species using a python script. All the genes aligned by MAFFT and used RAxML v8.1.24 [80] to conduct maximum likelihood (ML) analyses with the GTR + I + G model. The best scoring ML tree was obtained with 1000 bootstrap replicates. Then, we estimated divergence times of the plastome dataset, using an approximate likelihood method as implemented in MCMCtree (in PAML version 4) [81], with an independent relaxed clock and birth–death sampling [82]. The divergence between Salicaceae *s.l.* species and the outgroup, *Passiflora*, was assigned an age constraint of 87 to 97 Mya, as has been previously used [83,84,85,86].

## 5. Conclusions

We compared 24 plastomes, 20 newly sequenced and four other species sequences were collected from the NCBI GenBank, all representing 18 genera of the family Salicaceae *s.l.* The SSRs are considered effective molecular markers for plant species identification and population genetics. A total of 1798 SSRs were identified. Mono-nucleotide repeats were the most common with 1455 repeats accounting for about 80.92% of the total. Most of repeats were located in the non-coding region. We also identified five other types of repeats, including 1750 tandems, 434 forward, 407 palindromic, 86 reverse, and 30 complementary repeats. The species in Salicaceae *s.l*. have a conserved plastid genome. Each plastome presented a typical quadripartite structure and varied in size due to the expansion and contraction of the IR boundary, lacking major structural variations, but we identified six divergence hotspot regions (*matK-trnQ-UUG*, *trnS-GCU-trnG-UCC*, *psbZ-trnG-GCC*, *ndhF-trnL-UAG*, *psbE-petL*, and *ycf1+ndhF*). We obtained phylogenetic relationships in 18 genera of Salicaceae *s.l.* and the 26 species formed a highly supported lineage. *Casearia* was identified as the basal clade. The divergence time between Salicaceae *s.l.* and the outgroup was estimated as ~93 Mya, and *Salix* and *Populus* diverged around 34 Mya, consistent with previously reported times. This study demonstrates the potential of plastid genome to address the genus relationship of Salicaceae *s.l.* This data will contribute to further understanding of the phylogenetic relationships among Salicaceae *s.l.*

## Figures and Tables

**Table 1:** Characteristics of plastid genomes of 26 species.

Species	No. of Total Genes	Genome Size (bp)	LSC Length (bp)	SSC Length (bp)	IR Length (bp)	GC Content (%)	Protein-Coding Genes	No. of tRNAs Genes	No. of rRNAs Genes	Genes with Introns	GenBank Accesion Number
Populus euphratica *	131	157,839	85,858	16,649	27,666	36.5	86(8)	37(7)	8(4)	20(5)	MK267314
Salix interior *	127	156,620	85,979	16,305	27,168	37.0	80(5)	37(7)	8(4)	21(5)	NC_024681
*Bennettiodendron leprosipes*	131	157,872	85,643	16,439	27,895	36.7	86(8)	37(7)	8(4)	21(5)	MK281360
*Bennettiodendron brevipes*	131	158,605	86,350	16,501	27,877	36.7	86(8)	37(7)	8(4)	21(5)	MK046729
Idesia polycarpa Maxim *	130	157,017	84,787	16,512	27859	36.7	86(8)	36(7)	8(4)	19(5)	NC_032060
*Olmediella betschleriana*	131	158,171	85,783	16,588	27,900	36.7	85(8)	37(7)	8(4)	20(5)	MK044831
*Carrierea calycina*	131	158,085	84,663	17,570	27,926	36.8	86(8)	37(7)	8(4)	21(5)	MK263737
*Itoa orientalis*	131	157,875	84,495	17,742	27,819	36.7	86(8)	37(7)	8(4)	21(5)	MN078146
Poliothyrsis sinensis *	129	156,680	84,793	16,605	27641	36.8	85(8)	37(7)	8(4)	19(5)	NC_037412
*Flacourtia inermis*	131	155,535	83,706	16,577	27,626	36.9	86(8)	37(7)	8(4)	21(5)	MN078138
*Flacourtia ramontchii*	131	155,144	83,391	16,591	27,581	36.9	86(8)	37(7)	8(4)	21(5)	MN078145
*Flacourtia rukam*	131	155,582	83,854	16,598	27,565	36.9	86(8)	37(7)	8(4)	21(5)	MK281365
*Xylosma congestum*	131	155,986	84,180	16,650	27,578	36.8	86(8)	37(7)	8(4)	21(5)	MN078135
*Scolopia chinensis*	131	157,292	84,464	17,716	27,556	36.8	86(8)	37(7)	8(4)	21(5)	MN078144
*Scolopia saeva*	131	155,832	84,062	16,436	27,667	36.9	86(8)	37(7)	8(4)	21(5)	MN078143
*Dovyalis caffra*	131	155,182	83,593	16,683	27,453	37.0	86(8)	37(7)	8(4)	21(5)	MN078137
*Homalium racemosum*	131	156,961	85,818	16,547	27,298	36.6	86(8)	37(7)	8(4)	21(5)	MN078136
*Homalium cochinchinense*	131	155,339	83,701	16,566	27,536	36.8	86(8)	37(7)	8(4)	21(5)	MN078140
*Banara guianensis*	131	158,577	85,863	17,334	27,690	36.5	86(8)	37(7)	8(4)	21(5)	MH937752
*Prockia crucis*	131	157,406	85,622	17,024	27,380	36.7	86(8)	37(7)	8(4)	21(5)	MN078147
*Azara serrata*	131	158,306	85,059	17,889	27,697	36.5	86(8)	37(7)	8(4)	21(5)	MH719101
*Abatia parviflora*	131	156,426	84,800	16,724	27,451	36.5	86(8)	37(7)	8(4)	21(5)	MN078139
*Casearia decandra Jacq*	131	156,809	84,890	17,037	27,441	36.8	85(8)	37(7)	8(4)	21(5)	MN078142
*Casearia velutina*	131	156,008	84,446	17,220	27,171	36.8	85(8)	37(7)	8(4)	21(5)	MN078141
Passiflora laurifolia **	130	151,422	85,411	13,511	26,250	37.0	77(4)	37(7)	8(4)	20(5)	NC_038121
Passiflora ligularis **	128	150,827	85,471	13,502	25,927	37.0	77(4)	36(7)	8(4)	19(5)	NC_038122

Note: Numbers in brackets indicate genes duplicated in the IR regions. The species with * are previous published plastid genomes, and the species with ** are outgroups.

**Table 2:** List of genes present in the plastid genomes of twenty newly sequenced Salicaceae *s.l.* species.

Category	Gene	Type Gene						
**Self-replication**	**Ribosomal RNA**	*rrn16*	*rrn23*	*rrn4.5*	*rrn5*			
	**Transfer RNA**	trnA-UGC *	*trnfM-CAU*	trnI-GAU *	*trnM-CAU*	*trnR-ACG*	*trnS-UGA*	
		*trnC-GCA*	*trnG-GCC*	trnK-UUU *	*trnN-GUU*	*trnW-CCA*	*trnT-GGU*	
		*trnD-GUC*	trnG-UCC *	*trnL-CAA*	*trnY-GUA*	*trnR-UCU*	*trnT-UGU*	
		*trnE-UUC*	*trnH-GUG*	trnL-UAA *	*trnP-UGG*	*trnS-GCU*	*trnV-GAC*	
		*trnF-GAA*	*trnI-CAU*	*trnL-UAG*	*trnQ-UUG*	*trnS-GGA*	trnV-UAC *	
	**Small ribosomal units**	*rps11*	rps12 *	*rps14*	*rps15*	*rps18*		
		*rps19*	*rps2*	*rps3*	*rps4*	*rps7*	*rps8*	
	**Large ribosomal units**	*rpl14*	rpl16 *	rpl2 *	*rpl20*	*rpl22*	*rpl23*	
		*rpl33*	*rpl36*					
	**RNA polymerase subunits**	*rpoA*	*rpoB*	rpoC1 *	*rpoC2*			
**Photosynthesis genes**	**NADH dehydrogenase**	ndhA *	ndhB*	*ndhC*	*ndhD*	*ndhE*	*ndhF*	
		*ndhG*	*ndhH*	*ndhI*	*ndhJ*	*ndhK*		
	**photosystem I**	*psaA*	*psaB*	*psaC*	*psaI*	*psaJ*	ycf3 *	*ycf4*
	**photosystem II**	*psbA*	*psbB*	*psbC*	*psbD*	*psbE*	*psbF*	*psbH*
		*psbI*	*psbJ*	*psbK*	*psbL*	*psbM*	*psbN*	*psbT*
		*psbZ*						
	**cytochrome b/f complex**	*petA*	petB *	*petD*	*petG*	*petL*	*petN*	
	**ATP synthase**	*atpA*	*atpB*	*atpE*	atpF*	*atpH*	*atpI*	
	**Large subunit of rubisco**	*rbcL*						
**Other genes**	**Maturase**	*matK*						
	**Protease**	clpP *						
	**Acetyl-CoA-carboxylase subunit**	*accD*						
	**Envelope membrane protein**	*cemA*						
	**Component of TIC complex**	*ycf1*						
	**c-type cytochrome synthesis**	*ccsA*						
**Unknown**	**hypothetical genes reading frames**	*ycf2*						

Notes: the * symbols indicate genes with intron(s).

## Supplementary Materials

Supplementary materials can be found at https://www.mdpi.com/1422-0067/20/15/3788/s1.
