# Supplementary material for: Intergeneric Relationships within the Family Salicaceae s.l. Based on Plastid Phylogenomics
Source: Int J Mol Sci. 2019 Aug 2;20(15):3788. doi: 10.3390/ijms20153788 (PMC6696080; doi:10.3390/ijms20153788)
Supplement: Supplementary file 1 [file ijms-20-03788-s001.zip › Supplementary/Figure S1.pdf]

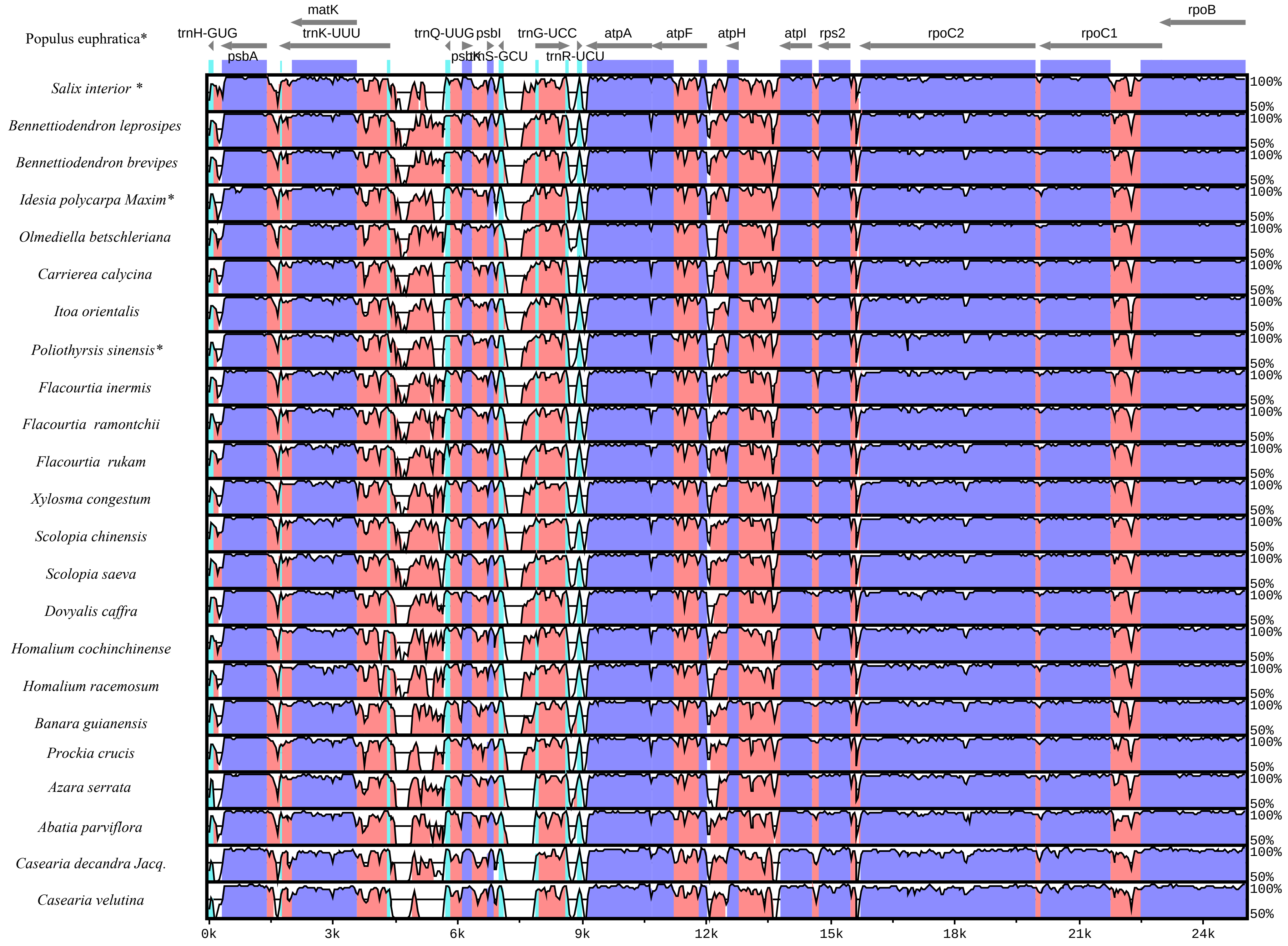
Populus euphratica*
matK
rpoB
trnH-GUG
trnK-UUU
trnQ-UUG
psbI
trnG-UCC
atpA
atpF
atpH
atpI
rps2
rpoC2
rpoC1
psbA
trnS-GCU
trnR-UCU
Salix interior *
Bennettiodendron leprosipes
Bennettiodendron brevipes
Idesia polycarpa Maxim*
Olmediella betschleriana
Carrierea calycina
Itoa orientalis
Poliothyrsis sinensis*
Flacourtia inermis
Flacourtia ramontchii
Flacourtia rukam
Xylosma congestum
Scolopia chinensis
Scolopia saeva
Dovyalis caffra
Homalium cochinchinense
Homalium racemosum
Banara guianensis
Prockia crucis
Azara serrata
Abatia parviflora
Casearia decandra Jacq.
Casearia velutina
100%
50%
0k
3k
6k
9k
12k
15k
18k
21k
24k

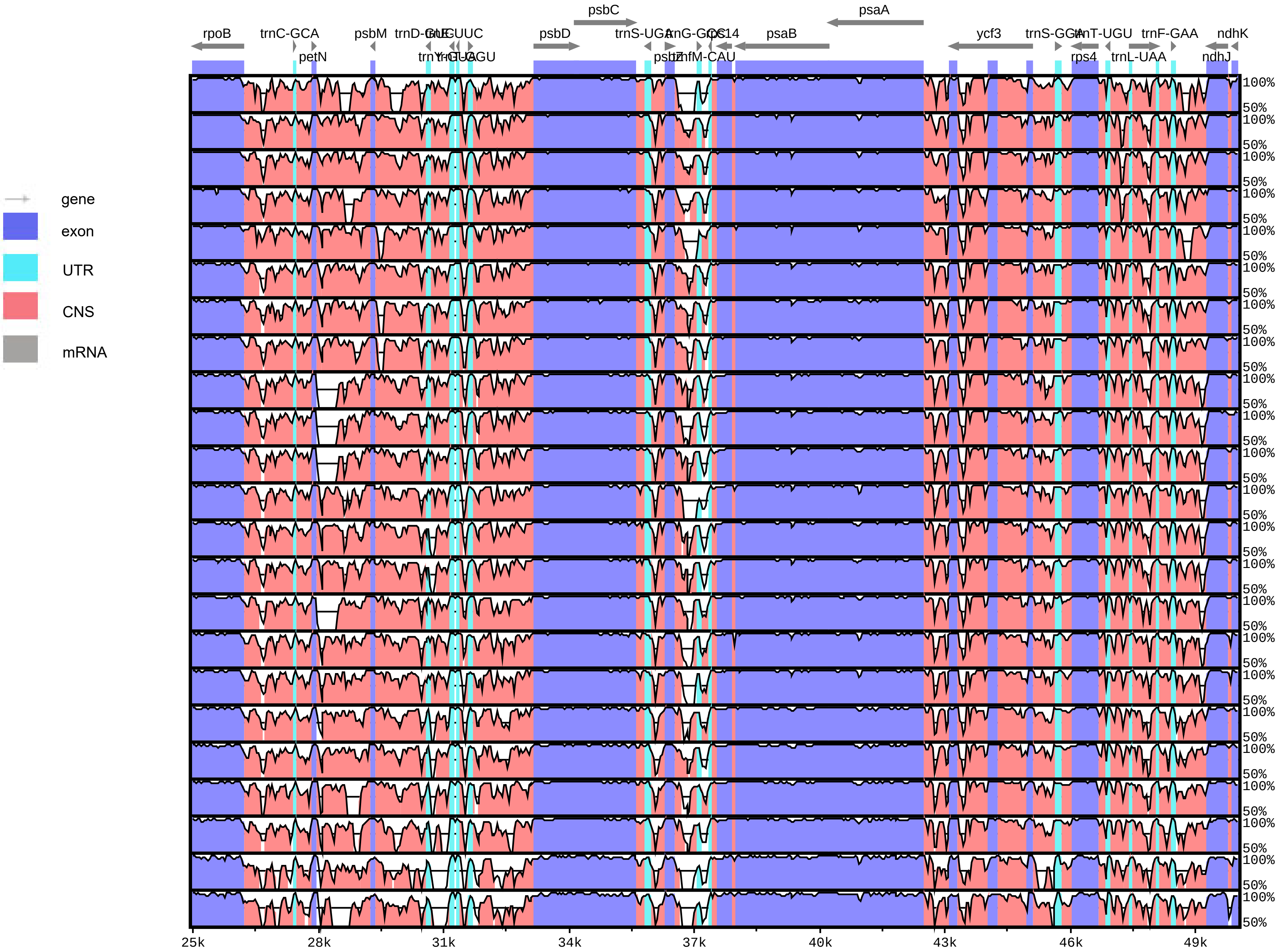

gene
exon
UTR
CNS
mRNA
rpoB
trnC-GCA
petN
psbM
psbD
psbC
trnS-UGA
rps14
psaB
psaA
ycf3
rps4
trnT-UGU
trnL-UAA
trnF-GAA
ndhJ
ndhK
100%
50%
25k
28k
31k
34k
37k
40k
43k
46k
49k

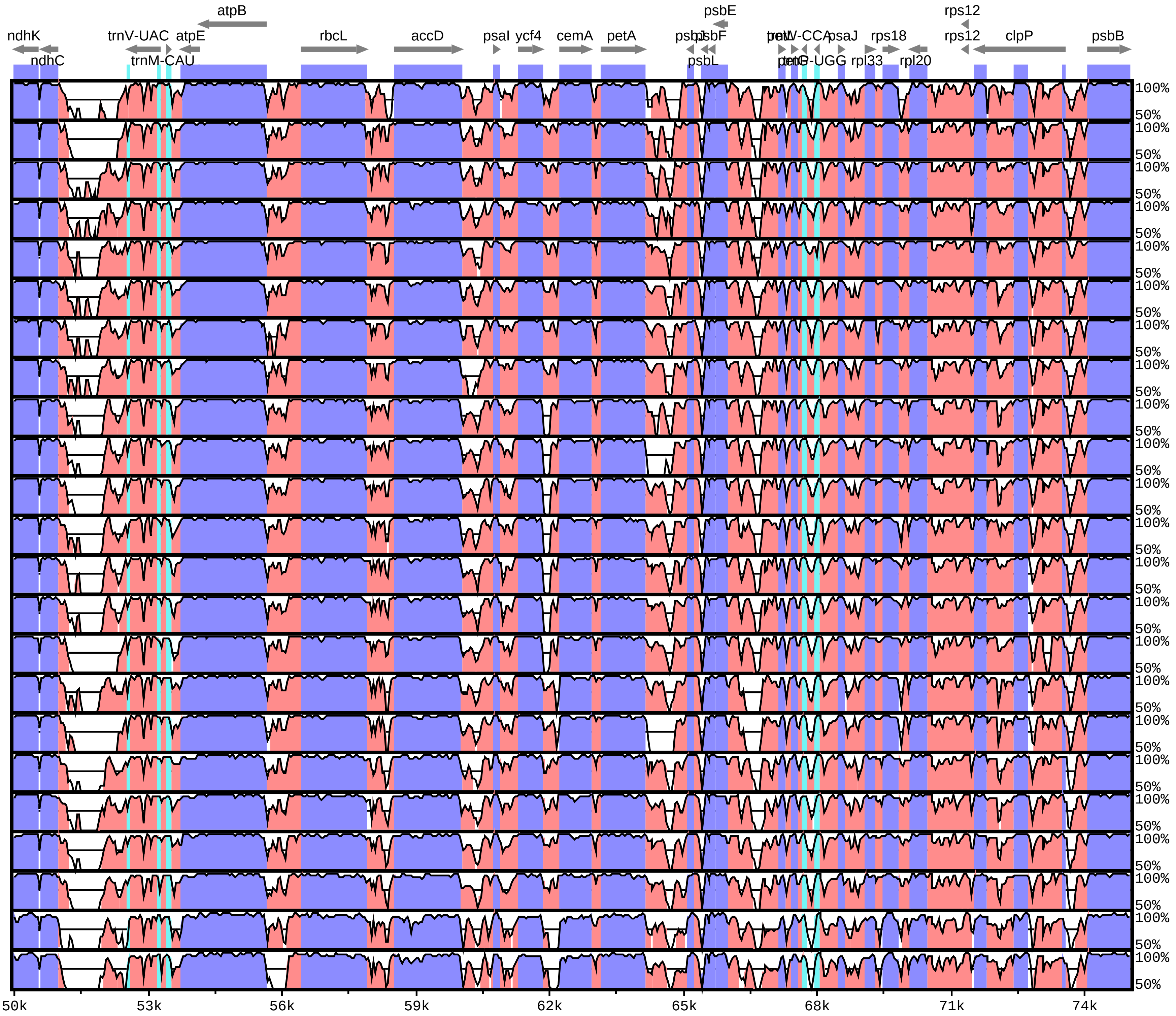
atpB
psbE
rps12
ndhK
trnV-UAC
atpE
rbcL
accD
psaI
ycf4
cemA
petA
psbF
psaJ
rps18
rps12
clpP
psbB
ndhC
trnM-CAU
psbL
trnP-UGG
rpl33
rpl20
100%
50%
50k
53k
56k
59k
62k
65k
68k
71k
74k

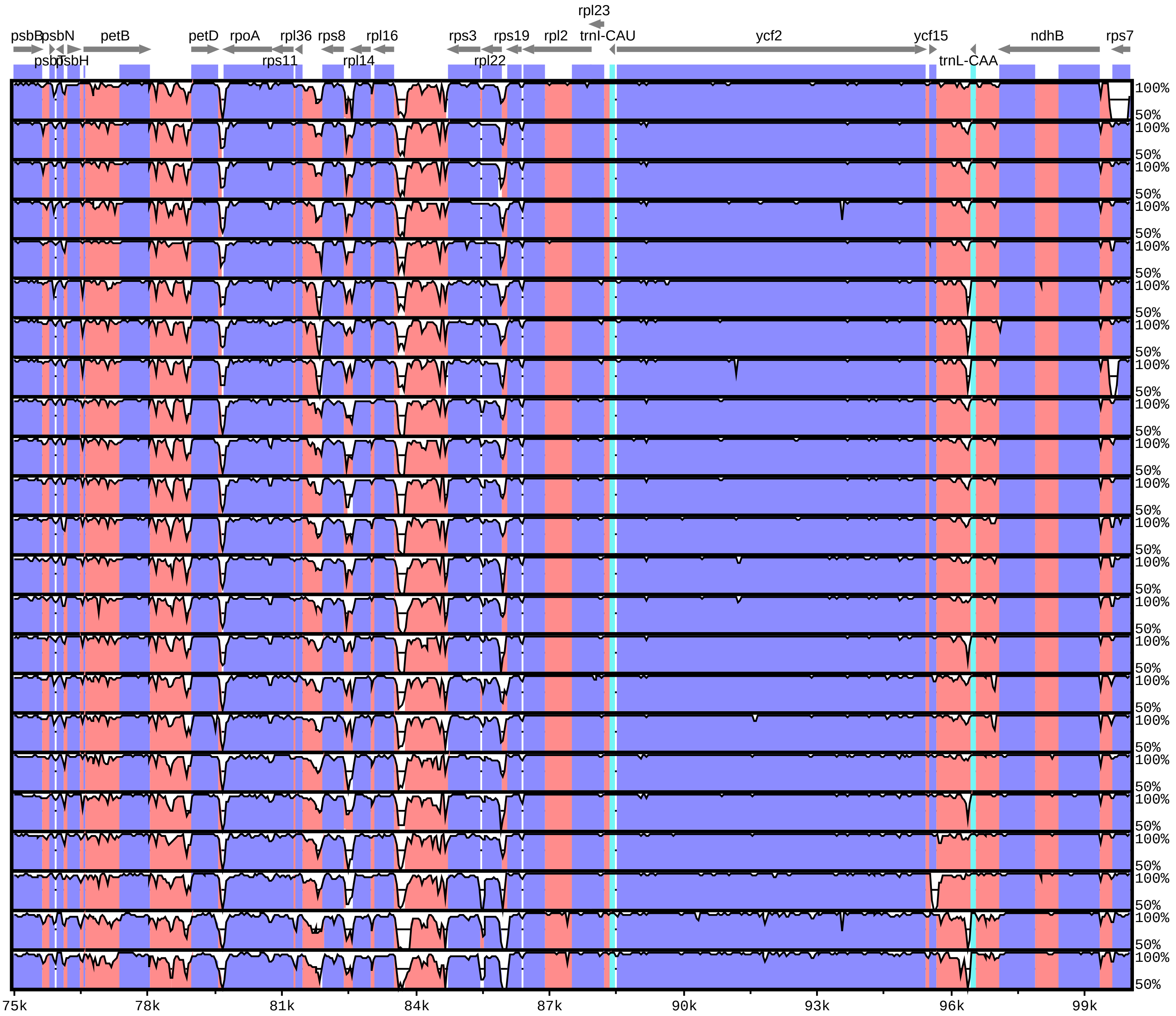

rpl23
psbB
psbN
petB
petD
rpoA
rpl36
rps8
rpl16
rps3
rps19
rpl2
trnI-CAU
ycf2
ycf15
ndhB
rps7
psbT
psbH
rps11
rpl14
rpl22
trnL-CAA
100%
50%
75k
78k
81k
84k
87k
90k
93k
96k
99k

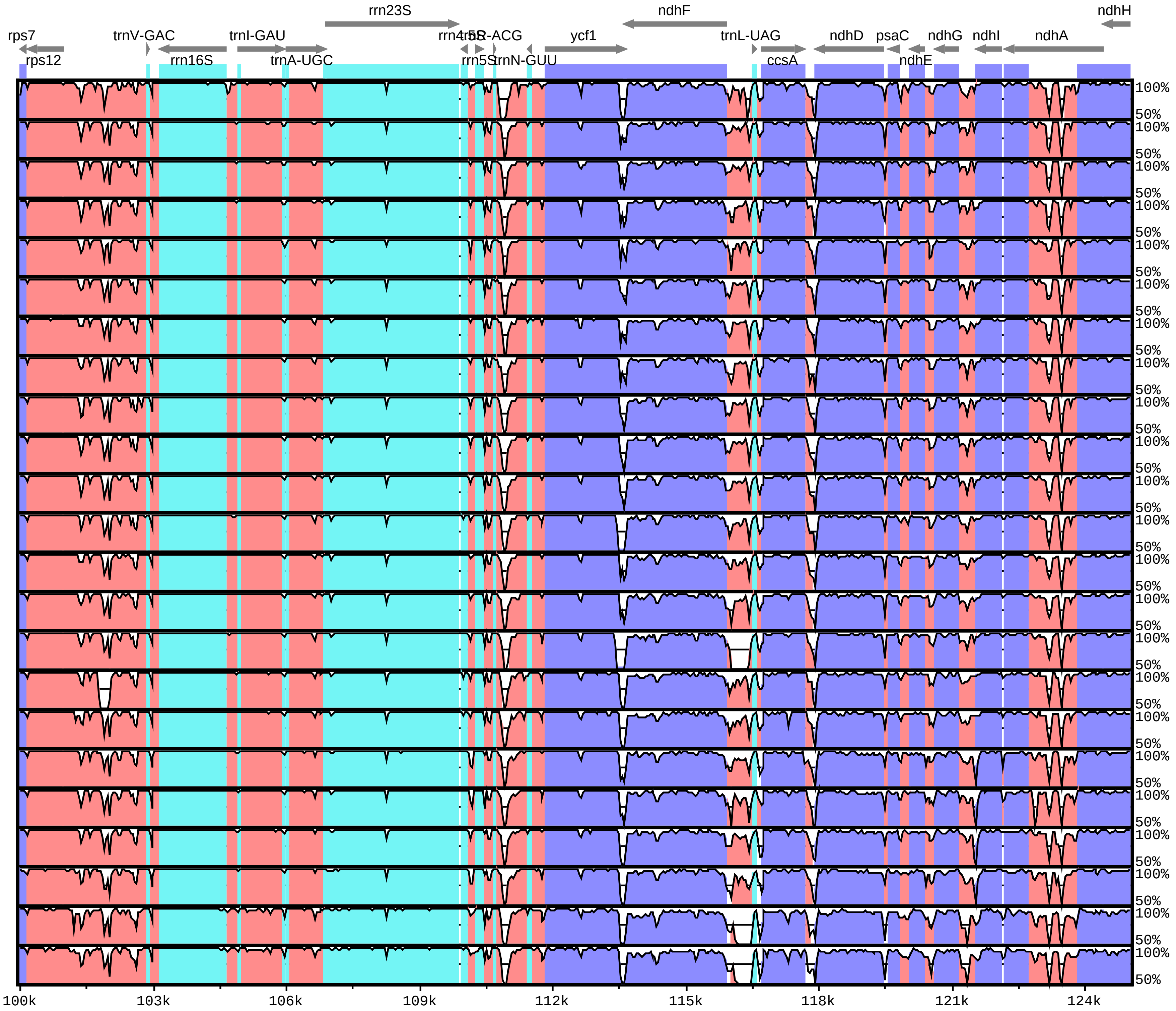

rrn23S
ndhF
ndhH
rps7
trnV-GAC
trnI-GAU
rrn4.5S
trnR-ACG
ycf1
trnL-UAG
ndhD
psaC
ndhG
ndhI
ndhA
rps12
rrn16S
trnA-UGC
rrn5S
trnN-GUU
ccsA
ndhE
100%
50%
100k
103k
106k
109k
112k
115k
118k
121k
124k

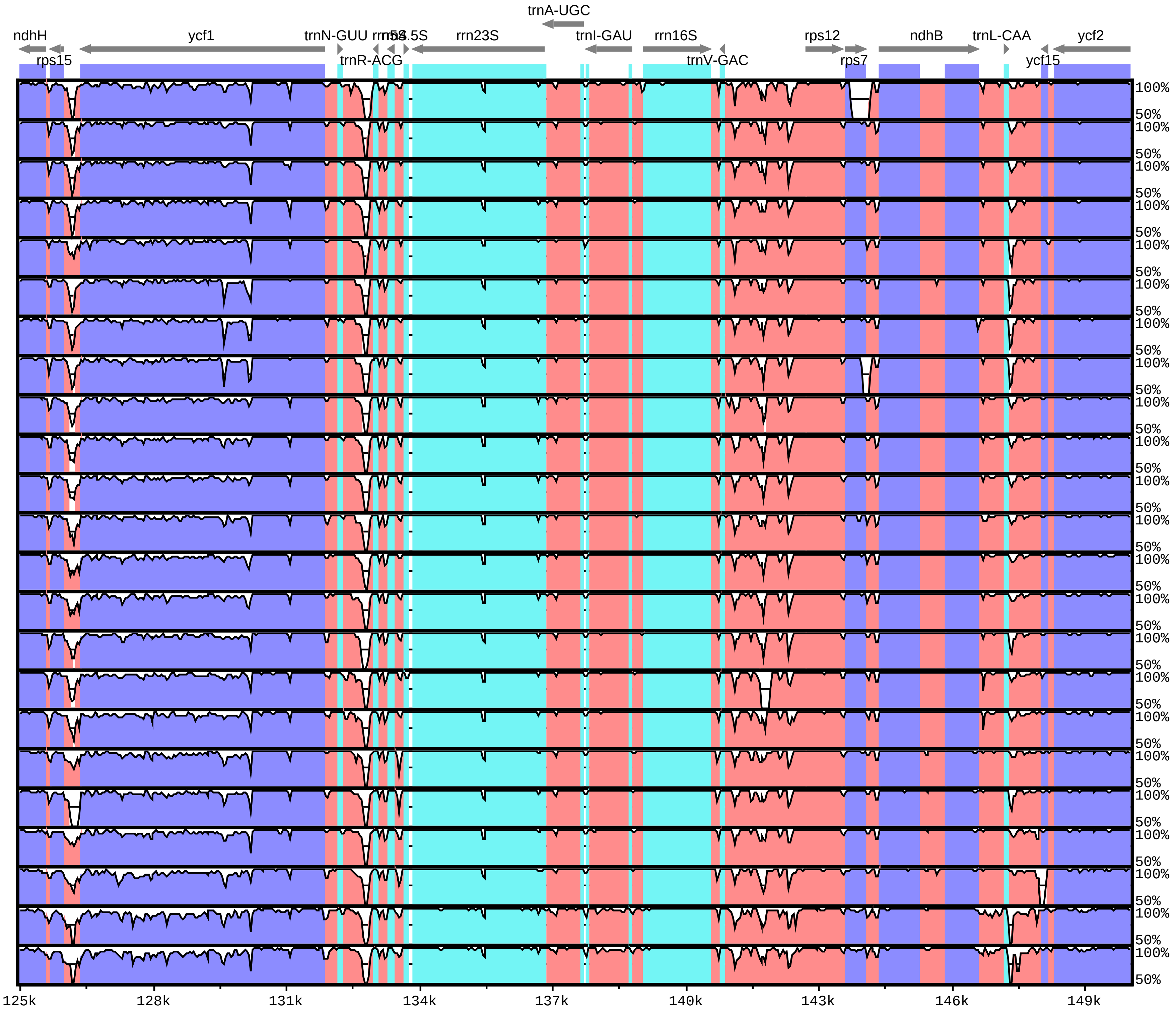
trnA-UGC
ndhH
ycf1
trnN-GUU
rrn5
rrn4.5S
rrn23S
trnI-GAU
rrn16S
rps12
ndhB
trnL-CAA
ycf2
rps15
trnR-ACG
trnV-GAC
rps7
ycf15
100%
50%
125k
128k
131k
134k
137k
140k
143k
146k
149k

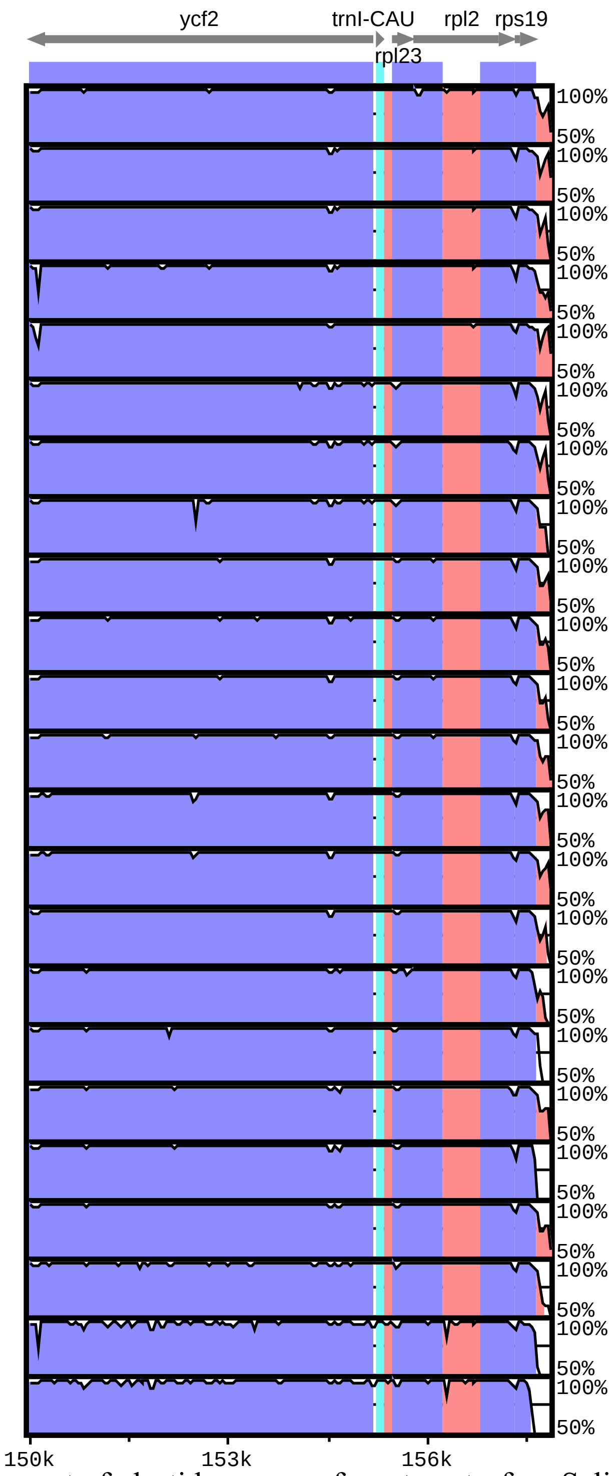

Figure S1. Sequence alignment of plastid genomes from twenty-four Salicaceae *s.l.* species. Sequences of plastid genomes were aligned and compared using the mVISTA program. The horizontal axis(x) indicates the coordinates within the plastid genome. The vertical scale (y axis) indicates the percentage identity, ranging from 50 to 100%. The grey arrows indicates the direction of each gene. Purple bars represent exons, orange bars show conserved non-coding sequences.
